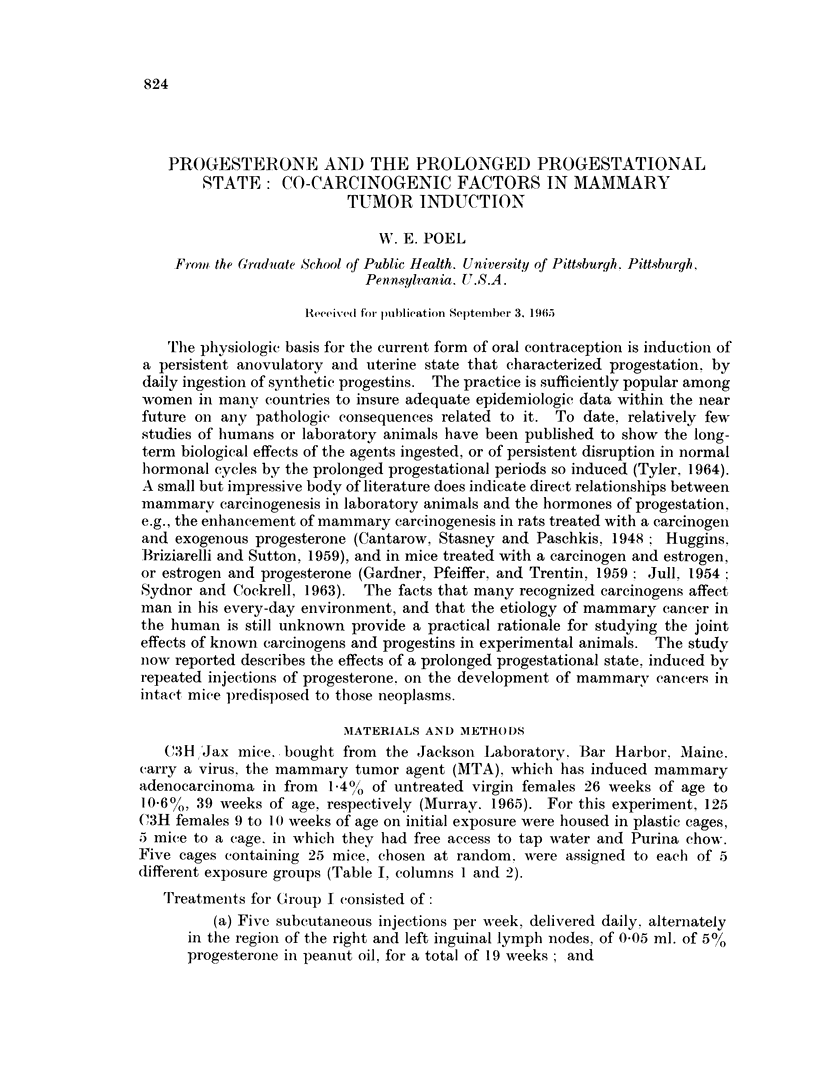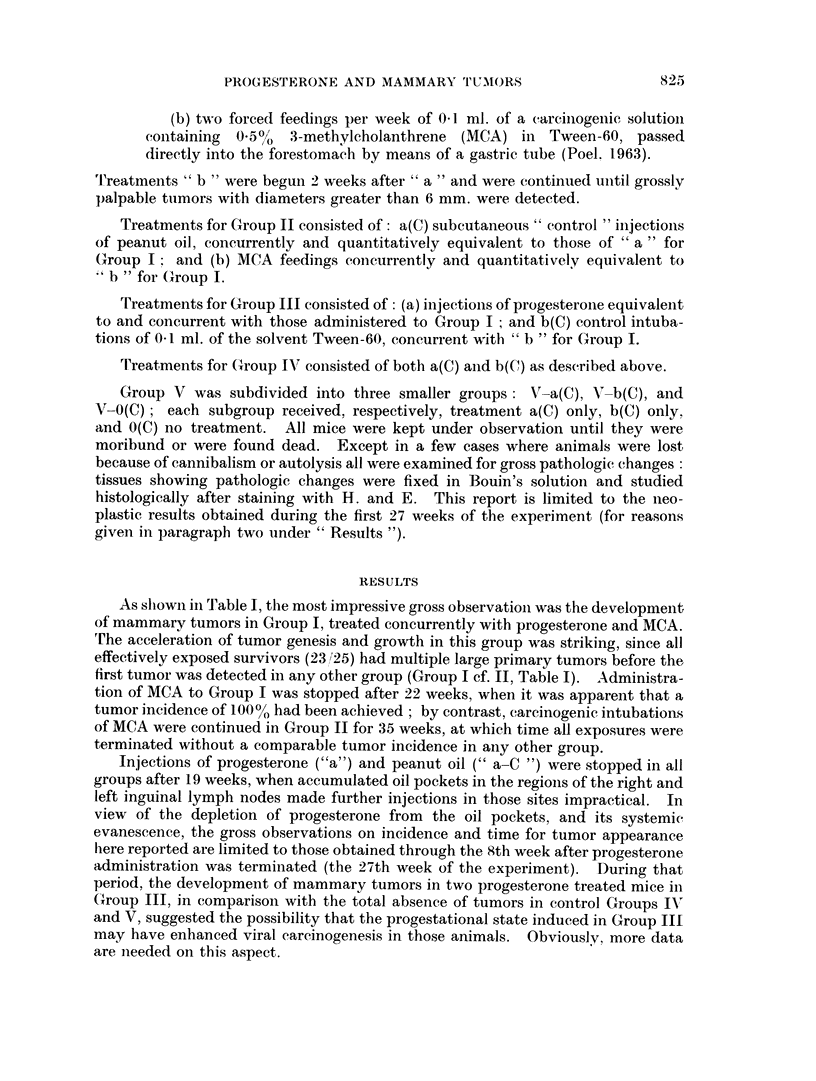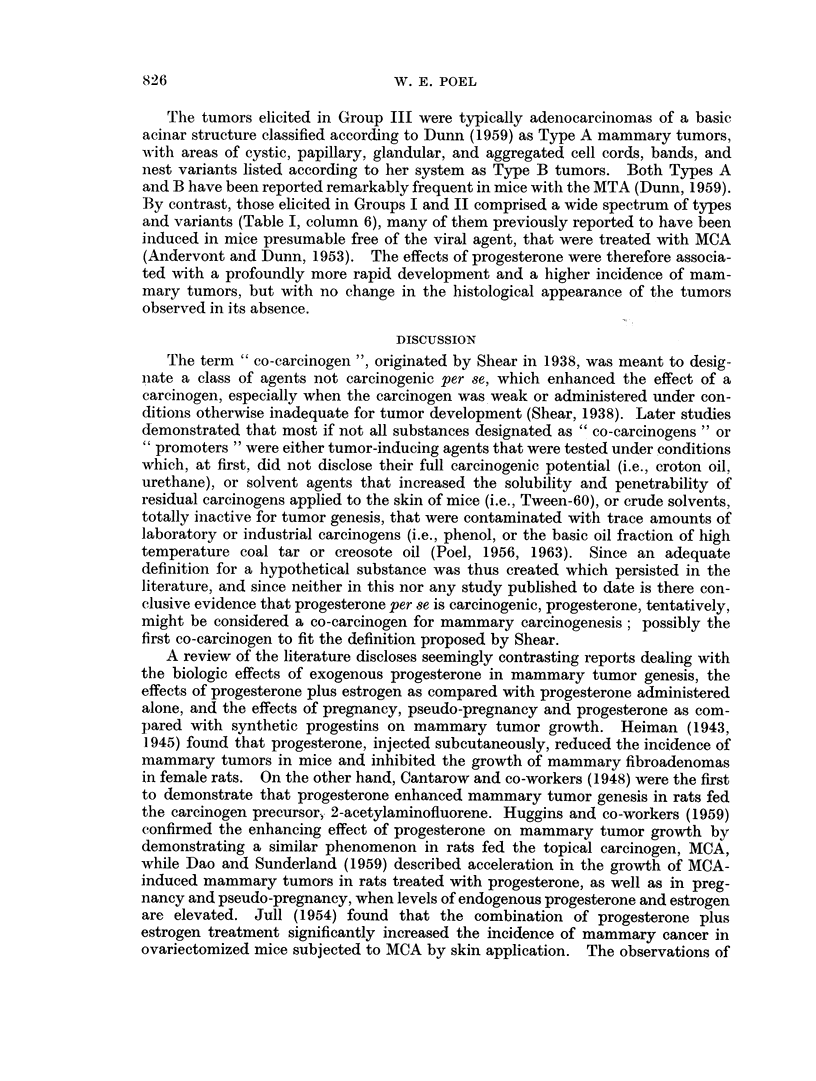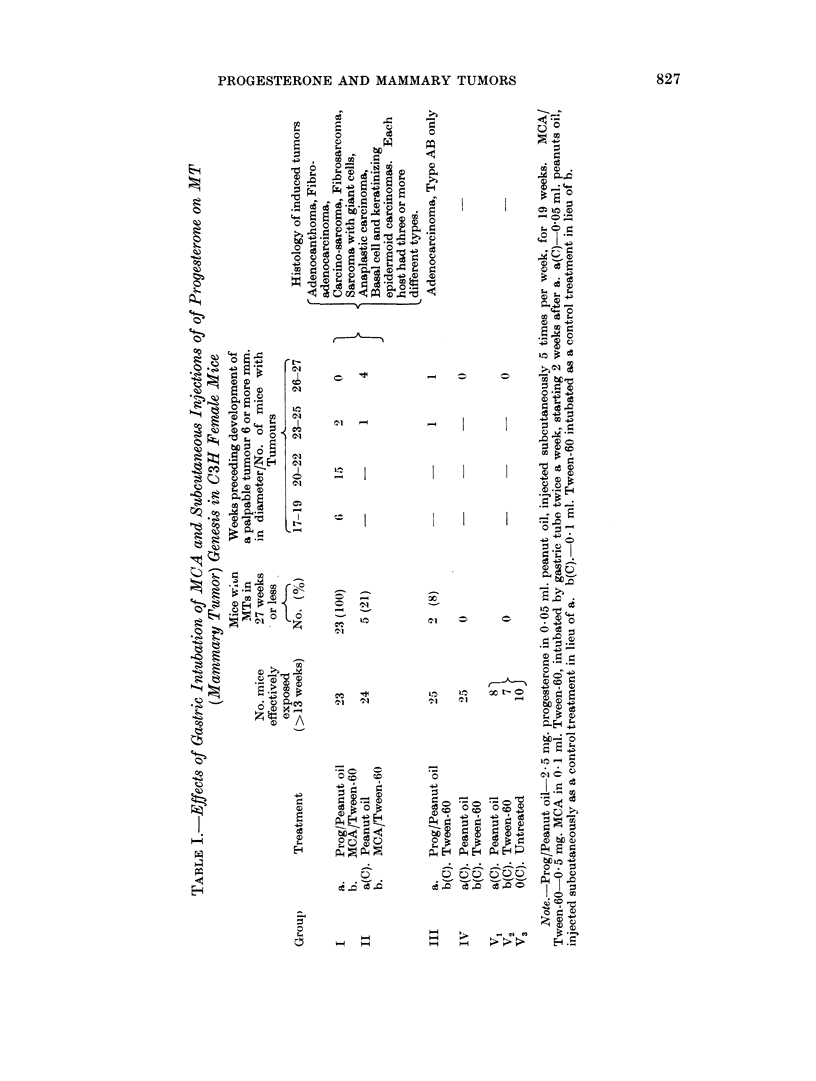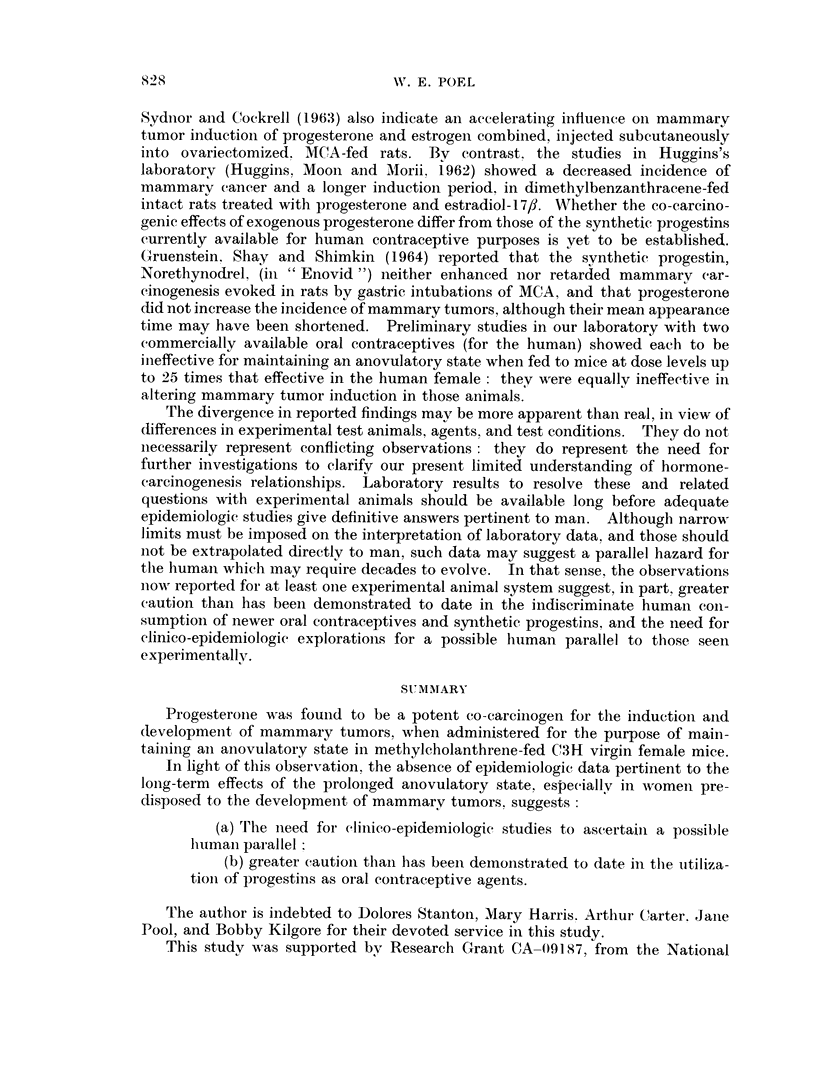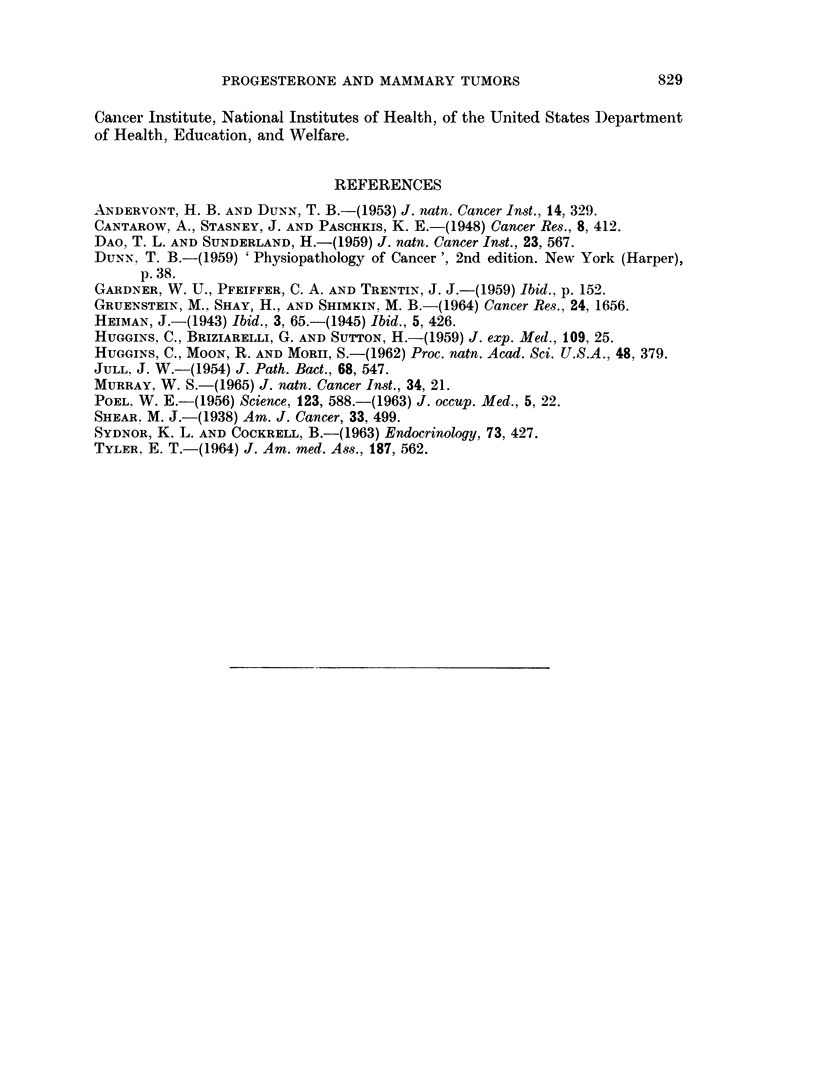# Progesterone and the prolonged progestational state: co-carcinogenic factors in mammary tumor induction.

**DOI:** 10.1038/bjc.1965.95

**Published:** 1965-12

**Authors:** W. E. Poel


### PROGESTERONE AND THE PROLONGED PROGESTATIONAL STATE: CO-CARCINOGENIC FACTORS IN MAMMARY TUMOR INDUCTION

W. E. POEL

*From the Graduate School of Public Health, University of Pittsburgh, Pittsburgh, Pennsylvania, U.S.A.*

Received for publication September 3, 1965

The physiologic basis for the current form of oral contraception is induction of a persistent anovulatory and uterine state that characterized progestation, by daily ingestion of synthetic progestins. The practice is sufficiently popular among women in many countries to insure adequate epidemiologic data within the near future on any pathologic consequences related to it. To date, relatively few studies of humans or laboratory animals have been published to show the long-term biological effects of the agents ingested, or of persistent disruption in normal hormonal cycles by the prolonged progestational periods so induced (Tyler, 1964). A small but impressive body of literature does indicate direct relationships between mammary carcinogenesis in laboratory animals and the hormones of progestation, e.g., the enhancement of mammary carcinogenesis in rats treated with a carcinogen and exogenous progesterone (Cantarow, Stasney and Paschkis, 1948; Huggins, Briziarelli and Sutton, 1959), and in mice treated with a carcinogen and estrogen, or estrogen and progesterone (Gardner, Pfeiffer, and Trentin, 1959; Jull, 1954; Sydnor and Cockrell, 1963). The facts that many recognized carcinogens affect man in his every-day environment, and that the etiology of mammary cancer in the human is still unknown provide a practical rationale for studying the joint effects of known carcinogens and progestins in experimental animals. The study now reported describes the effects of a prolonged progestational state, induced by repeated injections of progesterone, on the development of mammary cancers in intact mice predisposed to those neoplasms.

### MATERIALS AND METHODS

C3H/Jax mice, bought from the Jackson Laboratory, Bar Harbor, Maine, carry a virus, the mammary tumor agent (MTA), which has induced mammary adenocarcinoma in from 1·4% of untreated virgin females 26 weeks of age to 10·6%, 39 weeks of age, respectively (Murray, 1965). For this experiment, 125 C3H females 9 to 10 weeks of age on initial exposure were housed in plastic cages, 5 mice to a cage, in which they had free access to tap water and Purina chow. Five cages containing 25 mice, chosen at random, were assigned to each of 5 different exposure groups (Table I, columns 1 and 2).

Treatments for Group I consisted of:

(a) Five subcutaneous injections per week, delivered daily, alternately in the region of the right and left inguinal lymph nodes, of 0·05 ml. of 5% progesterone in peanut oil, for a total of 19 weeks; and

(b) two forced feedings per week of 0·1 ml. of a carcinogenic solution containing 0·5% 3-methylcholanthrene (MCA) in Tween-60, passed directly into the forestomach by means of a gastric tube (Poel, 1963).

Treatments " b " were begun 2 weeks after " a " and were continued until grossly palpable tumors with diameters greater than 6 mm. were detected.

Treatments for Group II consisted of : a(C) subcutaneous " control " injections of peanut oil, concurrently and quantitatively equivalent to those of " a " for Group I ; and (b) MCA feedings concurrently and quantitatively equivalent to " b " for Group I.

Treatments for Group III consisted of : (a) injections of progesterone equivalent to and concurrent with those administered to Group I ; and b(C) control intubations of 0·1 ml. of the solvent Tween-60, concurrent with " b " for Group I.

Treatments for Group IV consisted of both a(C) and b(C) as described above.

Group V was subdivided into three smaller groups : V–a(C), V–b(C), and V–0(C) ; each subgroup received, respectively, treatment a(C) only, b(C) only, and 0(C) no treatment. All mice were kept under observation until they were moribund or were found dead. Except in a few cases where animals were lost because of cannibalism or autolysis all were examined for gross pathologic changes : tissues showing pathologic changes were fixed in Bouin's solution and studied histologically after staining with H. and E. This report is limited to the neoplastic results obtained during the first 27 weeks of the experiment (for reasons given in paragraph two under " Results ").

### RESULTS

As shown in Table I, the most impressive gross observation was the development of mammary tumors in Group I, treated concurrently with progesterone and MCA. The acceleration of tumor genesis and growth in this group was striking, since all effectively exposed survivors (23/25) had multiple large primary tumors before the first tumor was detected in any other group (Group I cf. II, Table I). Administration of MCA to Group I was stopped after 22 weeks, when it was apparent that a tumor incidence of 100% had been achieved ; by contrast, carcinogenic intubations of MCA were continued in Group II for 35 weeks, at which time all exposures were terminated without a comparable tumor incidence in any other group.

Injections of progesterone ("a") and peanut oil (" a–C ") were stopped in all groups after 19 weeks, when accumulated oil pockets in the regions of the right and left inguinal lymph nodes made further injections in those sites impractical. In view of the depletion of progesterone from the oil pockets, and its systemic evanescence, the gross observations on incidence and time for tumor appearance here reported are limited to those obtained through the 8th week after progesterone administration was terminated (the 27th week of the experiment). During that period, the development of mammary tumors in two progesterone treated mice in Group III, in comparison with the total absence of tumors in control Groups IV and V, suggested the possibility that the progestational state induced in Group III may have enhanced viral carcinogenesis in those animals. Obviously, more data are needed on this aspect.

The tumors elicited in Group III were typically adenocarcinomas of a basic acinar structure classified according to Dunn (1959) as Type A mammary tumors, with areas of cystic, papillary, glandular, and aggregated cell cords, bands, and nest variants listed according to her system as Type B tumors. Both Types A and B have been reported remarkably frequent in mice with the MTA (Dunn, 1959). By contrast, those elicited in Groups I and II comprised a wide spectrum of types and variants (Table I, column 6), many of them previously reported to have been induced in mice presumable free of the viral agent, that were treated with MCA (Andervont and Dunn, 1953). The effects of progesterone were therefore associated with a profoundly more rapid development and a higher incidence of mammary tumors, but with no change in the histological appearance of the tumors observed in its absence.

### DISCUSSION

The term " co-carcinogen ", originated by Shear in 1938, was meant to designate a class of agents not carcinogenic *per se*, which enhanced the effect of a carcinogen, especially when the carcinogen was weak or administered under conditions otherwise inadequate for tumor development (Shear, 1938). Later studies demonstrated that most if not all substances designated as " co-carcinogens " or " promoters " were either tumor-inducing agents that were tested under conditions which, at first, did not disclose their full carcinogenic potential (i.e., croton oil, urethane), or solvent agents that increased the solubility and penetrability of residual carcinogens applied to the skin of mice (i.e., Tween-60), or crude solvents, totally inactive for tumor genesis, that were contaminated with trace amounts of laboratory or industrial carcinogens (i.e., phenol, or the basic oil fraction of high temperature coal tar or creosote oil (Poel, 1956, 1963). Since an adequate definition for a hypothetical substance was thus created which persisted in the literature, and since neither in this nor any study published to date is there conclusive evidence that progesterone *per se* is carcinogenic, progesterone, tentatively, might be considered a co-carcinogen for mammary carcinogenesis ; possibly the first co-carcinogen to fit the definition proposed by Shear.

A review of the literature discloses seemingly contrasting reports dealing with the biologic effects of exogenous progesterone in mammary tumor genesis, the effects of progesterone plus estrogen as compared with progesterone administered alone, and the effects of pregnancy, pseudo-pregnancy and progesterone as compared with synthetic progestins on mammary tumor growth. Heiman (1943, 1945) found that progesterone, injected subcutaneously, reduced the incidence of mammary tumors in mice and inhibited the growth of mammary fibroadenomas in female rats. On the other hand, Cantarow and co-workers (1948) were the first to demonstrate that progesterone enhanced mammary tumor genesis in rats fed the carcinogen precursor, 2-acetylaminofluorene. Huggins and co-workers (1959) confirmed the enhancing effect of progesterone on mammary tumor growth by demonstrating a similar phenomenon in rats fed the topical carcinogen, MCA, while Dao and Sunderland (1959) described acceleration in the growth of MCA-induced mammary tumors in rats treated with progesterone, as well as in pregnancy and pseudo-pregnancy, when levels of endogenous progesterone and estrogen are elevated. Jull (1954) found that the combination of progesterone plus estrogen treatment significantly increased the incidence of mammary cancer in ovariectomized mice subjected to MCA by skin application. The observations of

Sydnor and Cockrell (1963) also indicate an accelerating influence on mammary tumor induction of progesterone and estrogen combined, injected subcutaneously into ovariectomized, MCA-fed rats. By contrast, the studies in Huggins's laboratory (Huggins, Moon and Morii, 1962) showed a decreased incidence of mammary cancer and a longer induction period, in dimethylbenzanthracene-fed intact rats treated with progesterone and estradiol-17$\beta$. Whether the co-carcinogenic effects of exogenous progesterone differ from those of the synthetic progestins currently available for human contraceptive purposes is yet to be established. Gruenstein, Shay and Shimkin (1964) reported that the synthetic progestin, Norethynodrel, (in " Enovid ") neither enhanced nor retarded mammary carcinogenesis evoked in rats by gastric intubations of MCA, and that progesterone did not increase the incidence of mammary tumors, although their mean appearance time may have been shortened. Preliminary studies in our laboratory with two commercially available oral contraceptives (for the human) showed each to be ineffective for maintaining an anovulatory state when fed to mice at dose levels up to 25 times that effective in the human female : they were equally ineffective in altering mammary tumor induction in those animals.

The divergence in reported findings may be more apparent than real, in view of differences in experimental test animals, agents, and test conditions. They do not necessarily represent conflicting observations : they do represent the need for further investigations to clarify our present limited understanding of hormone-carcinogenesis relationships. Laboratory results to resolve these and related questions with experimental animals should be available long before adequate epidemiologic studies give definitive answers pertinent to man. Although narrow limits must be imposed on the interpretation of laboratory data, and those should not be extrapolated directly to man, such data may suggest a parallel hazard for the human which may require decades to evolve. In that sense, the observations now reported for at least one experimental animal system suggest, in part, greater caution than has been demonstrated to date in the indiscriminate human consumption of newer oral contraceptives and synthetic progestins, and the need for clinico-epidemiologic explorations for a possible human parallel to those seen experimentally.

SUMMARY

Progesterone was found to be a potent co-carcinogen for the induction and development of mammary tumors, when administered for the purpose of maintaining an anovulatory state in methylcholanthrene-fed C3H virgin female mice.

In light of this observation, the absence of epidemiologic data pertinent to the long-term effects of the prolonged anovulatory state, especially in women predisposed to the development of mammary tumors, suggests :

(a) The need for clinico-epidemiologic studies to ascertain a possible human parallel ;

(b) greater caution than has been demonstrated to date in the utilization of progestins as oral contraceptive agents.

The author is indebted to Dolores Stanton, Mary Harris, Arthur Carter, Jane Pool, and Bobby Kilgore for their devoted service in this study.

This study was supported by Research Grant CA-09187, from the National

Cancer Institute, National Institutes of Health, of the United States Department of Health, Education, and Welfare.

### REFERENCES

ANDERVONT, H. B. AND DUNN, T. B.—(1953) *J. natn. Cancer Inst.*, **14**, 329.
CANTAROW, A., STASNEY, J. AND PASCHKIS, K. E.—(1948) *Cancer Res.*, **8**, 412.
DAO, T. L. AND SUNDERLAND, H.—(1959) *J. natn. Cancer Inst.*, **23**, 567.
DUNN, T. B.—(1959) 'Physiopathology of Cancer', 2nd edition. New York (Harper), p. 38.
GARDNER, W. U., PFEIFFER, C. A. AND TRENTIN, J. J.—(1959) *Ibid.*, p. 152.
GRUENSTEIN, M., SHAY, H., AND SHIMKIN, M. B.—(1964) *Cancer Res.*, **24**, 1656.
HEIMAN, J.—(1943) *Ibid.*, **3**, 65.—(1945) *Ibid.*, **5**, 426.
HUGGINS, C., BRIZIARELLI, G. AND SUTTON, H.—(1959) *J. exp. Med.*, **109**, 25.
HUGGINS, C., MOON, R. AND MORII, S.—(1962) *Proc. natn. Acad. Sci. U.S.A.*, **48**, 379.
JULL, J. W.—(1954) *J. Path. Bact.*, **68**, 547.
MURRAY, W. S.—(1965) *J. natn. Cancer Inst.*, **34**, 21.
POEL, W. E.—(1956) *Science*, **123**, 588.—(1963) *J. occup. Med.*, **5**, 22.
SHEAR, M. J.—(1938) *Am. J. Cancer*, **33**, 499.
SYDNOR, K. L. AND COCKRELL, B.—(1963) *Endocrinology*, **73**, 427.
TYLER, E. T.—(1964) *J. Am. med. Ass.*, **187**, 562.